# Associations between plasma tenofovir concentration and renal function markers in HIV-infected women

**DOI:** 10.4102/sajhivmed.v17i1.458

**Published:** 2016-07-28

**Authors:** Mwila Mulubwa, Malie Rheeders, Carla Fourie, Michelle Viljoen

**Affiliations:** 1 Centre of Excellence for Pharmaceutical Sciences (Pharmacen), Division of Pharmacology, North-West University, South Africa; 2 Hypertension in Africa Research Team (HART), School for Physiology, Nutrition and Consumer Science, North-West University, South Africa

## Abstract

**Background:**

Tenofovir disoproxil fumarate (TDF) has been associated with kidney tubular dysfunction and reduced renal function. Limited studies were performed in Europe and Asia that related plasma tenofovir (TFV) concentration with renal function; no such studies to date have been performed on Africans.

**Objective:**

To investigate the correlation between plasma tenofovir (TFV) concentration and certain renal function markers in HIV-infected women on TDF antiretroviral therapy (ART). These markers were also compared to a HIV-uninfected control group.

**Methods:**

HIV-infected women (*n* = 30) on TDF-based ART were matched with 30 controls for age and body mass index. Renal markers analysed were estimated glomerular filtration rate (eGFR), creatinine clearance (CrCl), serum creatinine, albuminuria, glucosuria, serum urea, serum uric acid, urine sodium and maximum tubular reabsorption of phosphate. Baseline eGFR and CrCl data were obtained retrospectively for the HIV-infected women. Plasma TFV was assayed using a validated HPLC-MS/MS method. Stepwise regression, Mann–Whitney test, unpaired and paired *t*-tests were applied in the statistical analyses.

**Results:**

TFV concentration was independently associated with albuminuria (adjusted *r^2^* = 0.339*; p* = 0.001) in HIV-infected women. In the adjusted (weight) analysis, eGFR (*p* = 0.038), CrCl (*p* = 0.032) and albuminuria (*p* = 0.048) were significantly higher in HIV-infected compared to the uninfected women, but eGFR was abnormally high in HIV-infected women. Both eGFR (*p* < 0.001) and CrCl (*p* = 0.008) increased from baseline to follow-up in HIV-infected women.

**Conclusion:**

Plasma TFV concentration was associated with increased albuminuria in HIV-infected women in this sub-study. Both eGFR and CrCl were increased in HIV-infected women from baseline. These findings should be confirmed in larger studies, and hyperfiltration in HIV-infected women warrants further investigation.

## Introduction

Tenofovir disoproxil fumarate (TDF) is a prodrug of tenofovir (TFV), a nucleotide reverse transcriptase inhibitor (NRTI). In combination with other antiretroviral drugs, TDF-based antiretroviral therapy (ART) is used as preferred effective first-line treatment for adults infected with the human immunodeficiency virus (HIV).^1^

TFV plasma binding is low with less than 1 and 7.2% bound in human plasma and serum, respectively. It is eliminated mainly by renal excretion through a combination of glomerular filtration and active tubular secretion via the apical multidrug resistance protein (MRP4) transporter.^2,3^ In many studies, treatment with TDF was associated with kidney tubular dysfunction and reduced renal function, with higher prevalence being associated with female gender and age between 40 and 50 years.^4,5,6^ The main target of toxicity is the proximal renal tubule, and in severe cases patients developed renal Fanconi syndrome.^7^ Studies have shown that albuminuria might be a more reliable marker for glomerular and proximal tubular dysfunction.^8^ The effect of TFV on glomerular function is less severe than renal tubular function.^6^ TFV nephrotoxicity leads to break down of solute transport characterised by urine wasting of solutes and proteins such as albumin normally reabsorbed in the proximal tubule.^7^ Genetic polymorphisms in proximal tubule transporters may predispose certain individuals to accumulate high intracellular TFV levels and could increase the risk of developing proximal tubular toxicity.^9^ Chronic kidney disease (CKD) is usually silent until later stages; thus, many patients with CKD are detected only shortly before the onset of symptomatic kidney failure.^10^ Furthermore, increased exposure to TDF is associated with a higher incidence of CKD.^10^ Co-administration of TDF with protease inhibitors (PIs) is associated with even greater declines in renal function.^11^

Despite the fact that TDF treatment had been associated with CKD, only a few studies ^12,13,14,15^ were performed in Europe and Asia that related plasma TFV concentration with renal function. In these studies, the marker of kidney function that was mostly investigated was estimated glomerular filtration rate (eGFR) or the presence of at least two of the following criteria: total daily excretion of phosphorus (> 1200 mg), fractional tubular absorption for phosphorus (< 0.80%), fractional excretion of uric acid (> 15%), β2 microglobulin of more than 1 mg/day and non-diabetic glucosuria (> 300 mg/day). There are no such studies to date that have been performed on Africans, and this information is lacking in South Africa.

The objective of this study was to determine the correlations between plasma TFV concentration and renal function markers in HIV-infected women receiving TDF-based ART and to assess changes in renal function from baseline (before TDF initiation). We also compared these markers with those in a matched comparative HIV-uninfected control group.

## Methods

This was a pilot cross-sectional sub-study within the Prospective Urban and Rural Epidemiology-South Africa (PURE-SA) study.^16^ PURE is a large longitudinal epidemiological study taking place in low-, middle- and high-income countries around the world and since 2005 in the North West province of South Africa. In total, 462 non-Caucasian women participated in this sub-study within the PURE-SA study in November 2012, April and August 2013 in the Tlokwe and Ganyesa districts of the North West province in South Africa. Thirty women that participated in the 2012–2013 study were HIV-infected and on full ART taking 300 mg TDF *nocte* (19:00–21:00, self-reported). They were matched with 30 HIV-uninfected women for age and body mass index (BMI) within the PURE-SA for this study.

Participants signed written informed consent forms for the PURE-SA and for this sub-study, respectively; the protocol was approved by the Research Ethics Committee of the North–West University (NWU-00016–10–A1) and North West Department of Health in 2013. During visits for the sub-study, participants were seen at NWU metabolic clinic (Potchefstroom Campus in the Tlokwe district) and at Sethlare Lodge (Ganyesa) on pre-arranged dates. Medication history was recorded by qualified pharmacists and assistants using a structured questionnaire.

Participants who had a medical history of renal disease, amputees or on known renal toxic medication (streptomycin, lithium, sulfadiazine, phenytoin, allopurinol, amphotericin B deoxycholate, methotrexate, statins and mesalazine) were excluded from this study. Concomitant medication use for hypertension and tuberculosis (rifampicin, hydrochlorothiazide, nifedipine and isoniazid) was acceptable if medical history was available.

### Sample analysis and outcome variables

Participants were asked to fast for at least 8 hours. Blood and spot urine samples for the participants were collected in the morning between 08:00 and 10:00 by a registered nurse. Serum creatinine (SCr), serum phosphate (SrP), serum uric acid, serum urea, glucosuria, urine creatinine (UCr), urine phosphate (UP), urine sodium (UNa) and urine albumin were analysed on a Cobas Integra 400 plus (Roche Switzerland). Plasma TFV was quantified by a validated high-performance liquid chromatography tandem mass spectrometry method^17^ for the HIV-infected participants on TDF-based ART. Accuracy and precision was less than 15% of the relative standard error. The mean linear regression coefficient (*r^2^*) of the calibration curves over the concentration range 12.5 ng/mL – 600 ng/mL was 0.9958 with lower limit of quantification (LLOQ) of 12.5 ng/mL. The extraction recovery was 96.9%.

The eGFR was calculated using the modification of diet in renal disease (MDRD)^18^ equation without inclusion of the black ethnicity factor for black South Africans as prediction is better without the ethnicity factor^19^:

$$\text{eGFR}(\text{mL}/\text{min}/1.73{\text{m}}^{2})=30 849\times {\text{SC}}_{\text{r}}{}^{-1.154}\times {\text{age}}^{-0.203}\times 0.742$$ [Eqn 1]

where SCr is in µmol/L.

The modified Cockroft–Gault (CG) formula^1,20^ was used to calculate creatinine clearance (CrCl) as follows:

CrCl(mL/min)=(140−age)×weight×0.85/SCr [Eqn 2]

where SCr is in µmol/L.

Maximum tubular reabsorption of phosphate (TmPO_4_/GFR) was calculated using the following formula^21^:

$${\text{TmPO}}_{4}/\text{GFR}=0.3\times \text{TRP}/(1-0.8\times \text{TRP})\times \text{SrP}$$ [Eqn 3]

where

TRP=1−(UP/SrP)×(SCr/UCr). [Eqn 4]

If TRP was 0.86 or less, then TmPO_4_/GFR was calculated with the formula:

$${\text{TmPO}}_{4}/\text{GFR}=\text{TRP}\times \text{SrP}$$ [Eqn 5]

Albuminuria was calculated as a ratio of urine albumin to urine creatinine (uACR)^22^:

albuminuria=urine albumin (mg/L)/urine creatinine (mmol/L) [Eqn 6]

Glomerular hyperfiltration was defined as eGFR > 150 mL/min/1.73 m^2^ for women.^23^

Baseline information on the weight prior to TDF initiation, SCr, duration of TDF ART and non-TDF ART regimens was recorded retrospectively from the clinic and hospital files of the institutions where these participants accessed their healthcare services.

### Statistical analysis

The selection of HIV-uninfected comparative group was performed by propensity score matching with the HIV-infected women. Propensity scores were estimated from a logistic multivariate regression model containing age and BMI as predictors used to model case and/or control membership. The selection of controls for the cases was performed automatically from the resulting propensity variable. The optimal match tolerance was 0.26, and sampling of controls was done without replacement.

All variables in the two groups were tested for approximate normality using the Shapiro–Wilk test and visual inspection of respective normal Q–Q plots. Median and interquartile range (IQR) values were computed for the description of variables.

Pearson’s correlations were performed between plasma TFV concentrations and renal function variables in HIV-infected women. Block stepwise linear regression analysis was performed to test for associations between variables within the HIV-infected group, adjusted for age and BMI.

The Mann–Whitney test or unpaired *t*-test was used to compare variables between the HIV-infected and the HIV-uninfected group if data were abnormally or normally distributed, respectively. Adjusted analyses for weight were performed using analysis of covariance (ANCOVA) and results were presented in Table 2. Associations between groups, stratified by TDF exposure (HIV-infected) and HIV-uninfected status controlling for age and BMI, were performed. CrCl and eGFR were further categorised and presented in Table 3.

A linear regression model was used to test for interactions. Paired *t*-tests were performed in the HIV-infected group to determine the mean change of CrCl, SCr and eGFR from baseline to follow-up time. A two-tailed significance testing level of 0.05 was used. All statistical analyses were performed using IBM^®^ SPSS^®^ Statistics software, version 22.

## Results

Participants’ characteristics are presented in Table 1. TFV could not be quantified in five of the 30 participants’ plasma samples. The median (IQR) TFV plasma concentration was 113 (74–139.4) ng/mL for the 25 HIV-infected participants. The lowest and highest plasma concentrations were 17.2 and 434.2 ng/mL, respectively.

**TABLE 1:** Characteristics of HIV-infected and uninfected women (control group).

Characteristics	HIV-infected	TDF exposed	%	HIV-uninfected	Control	*p*-value
Number of participants, (*n*)	30.0	-	-	30.0		-
Median age (IQR), years	52.0	49.0–59.0	-	57.0	53.7–63.3	0.008§
Median BMI (IQR), kg/m^2^	20.5	18.2–28.9	-	24.4	19.4–33.5	0.333
Median weight (IQR), kg	52.5	44.0–68.7	-	60.4	49.1–84.3	0.179
Median TDF ART exposure (months)	16.6	8.8–23.4†	-	-	-	-
Median non–TDF ART exposure (months)	24.5	16.8–43.8‡	-	-	-	-
Median time since HIV diagnosis (months)	31.0	22.0–53.5‡	-	-	-	-
**Other antiretroviral drugs in regimen:**						
Lamivudine	30.0	-	100.0	-	-	-
Efavirenz	30.0	-	100.0	-	-	-
**Concomitant medication:**						
Hydrochlorothiazide	1.0	-	3.3	None	-	-
Nifedipine	1.0	-	3.3	None	-	-
**Underlying disease:**						
Hypertension	2.0	-	6.7	None	-	-

† *n* = 24 (Four participants declined to provide consent to access their medical records, and information from two medical records was missing);

‡ *n* = 22 (Four participants declined to provide consent to access their medical records, and information from four medical records was missing);

§ The age was already adjusted for with BMI when selecting controls via propensity score matching.

### Plasma tenofovir concentration and renal function markers in HIV-infected women

**FIGURE 1:**
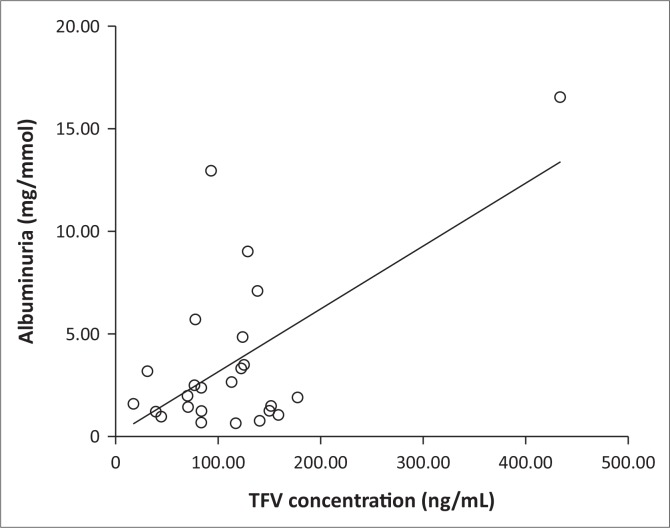
Regression plot of association between plasma tenofovir (TFV) concentration and albuminuria in HIV-infected women, age and BMI as fixed factors (adjusted *r*^2^ = 0.339; *p* = 0.001).

No significant correlations were found between plasma TFV concentration and eGFR, CrCl, TmPO_4_/GFR, SCr, UNa, serum urea or serum uric acid (*p* > 0.05). Nevertheless, a positive correlation was found between TFV plasma concentration and albuminuria (unadjusted *r* = 0.606; *p* = 0.001). Stepwise linear regression analysis was then performed to test for associations between TFV concentration and albuminuria with age and BMI as fixed factors. TFV concentration was independently associated with increased albuminuria (Figure 1).

In the unadjusted analysis, albuminuria (18 mg/mmol vs 8.8 mg/mmol; *p* = 0.028) and eGFR (126 mL/min/1.73 m^2^vs 105 mL/min/1.73 m^2^; *p* = 0.02) were significantly higher in the HIV-infected group compared to the matched HIV-uninfected group (Table 2). SCr (50 µmol/L vs 54 µmol/L; *p* = 0.028) and UNa (76 mmol/L vs 102 mmol/L; *p* = 0.048) were significantly lower in HIV-infected group compared to the HIV-uninfected group. No statistical significant difference in TmPO_4_/GFR, CrCl, serum urea and serum uric acid was found between the groups (*p* > 0.05).

**TABLE 2:** Comparison between mean values (and standard deviations) of renal function markers in HIV-infected (*n* = 30) and uninfected women (*n* = 30).

Variable	HIV-infected (TDF exposed)	HIV-uninfected (control)	*p*-value	Adjusted *p*-value§
SCr (µmol/L)	50.0 ± 20.3	54.0 ± 12.1	0.028†*	0.441
SrP (mmol/L)	1.05 ± 0.2	0.99 ± 0.16	0.204‡	-
Serum urea (mmol/L)	3.3 ± 1.7	3.50 ± 1.32	0.507‡	-
Serum uric acid (µmol/L)	278.7 ± 122	311 ± 99.6	0.265‡	-
Glucosuria (mmol/L)	0.12 ± 0.09	0.19 ± 0.27	0.59†	-
UCr (mmol/L)	4.3 ± 3.1	6.0 ± 5.8	0.145‡	-
UP (mmol/L)	5.4 ± 5.3	7.5 ± 9.4	0.524†	-
UNa (mmol/L)	75.5 ± 55.7	101.7 ± 42.3	0.048‡*	0.119
Albuminuria (mg/mmol)	18.0 ± 78.3	8.8 ± 27.9	0.028†*	0.048*
TmPO_4_/GFR	1.18 ± 0.24	1.15 ± 0.27	0.645‡	-
eGFR (mL/min/1.73 m^2^)	125.8 ± 39.6	105.0 ± 25.5	0.02‡*	0.047*
CrCl (mL/min)	112.5 ± 40.5	104.9 ± 43.0	0.486‡	0.048*
Urine albumin (mg/L)	33.9 ± 131.0	35.5 ± 112.4	0.126†	-

† *p*-value calculated from Mann–Whitney test;

‡ *p*-value calculated from unpaired *t*-test;

§ *p*-value resulting from ANCOVA adjusted for weight.

* *p* < 0.05.

ANCOVA adjusted for weight showed significantly higher CrCl (112.5 mL/min vs 104.9 mL/min; *p* = 0.048), eGFR (125.8 mL/min/1.73 m^2^vs 105 mL/min/1.73 m^2^; *p* = 0.047) and albuminuria (18 mg/mmol vs 8.8 mg/mmol; *p* = 0.048) in HIV-infected group compared to the matched HIV-uninfected control group.

A larger proportion of HIV-infected participants (33.3%) had abnormally higher eGFR (glomerular hyperfiltration, eGFR of > 150 mL/min/1.73 m^2^) compared to the HIV-uninfected (10%) control group. The numbers of participants in the respective categories are presented as percentages in Table 3.

**TABLE 3:** Percentage eGFR and CrCl categories in HIV-infected and uninfected women (*n* = 30).

Renal marker	HIV-infected		HIV-uninfected	
	
	*n*	%	*n*	%
eGFR <60 mL/min/1.73 m^2^	2	6.7	1	3.3
CrCl <60 mL/min	1	303.0	4	13.3
eGFR 60–150 mL/min/1.73 m^2^	18	60.0	26	86.7
CrCl 60–150 mL/min	25	83.3	22	73.3
eGFR > 150 mL/min/1.73 m^2^	10	33.3	3	10.0
CrCl > 150 mL/min	4	13.3	4	13.3

The linear regression analysis showed no significant interaction effect between weight and HIV-infected status (TDF exposure, *p* > 0.05).

### Retrospective changes in renal function markers (baseline and follow-up in HIV-infected women)

Baseline values for eGFR, SCr and CrCl were only available for 21 of the HIV-infected participants as information was missing or not included in their medical records. The eGFR significantly increased by 30.7% (+38.5 mL/min/1.73 m^2^, *p* < 0.001) from baseline (87 mL/min/1.73 m^2^ ± 29 mL/min/1.73 m^2^) to follow-up. CrCl increased by 25% (+26.5 mL/min, *p* = 0.008) from baseline (80 mL/min ± 28 mL/min) to follow-up. SCr decreased by 34.5% (-17.9 mmol/L, *p* = 0.017) from baseline (70 µmol/L ± 29 µmol/L) to follow-up. These changes occurred within a median duration of 16.6 months of TDF exposure.

## Discussion

In this study, uACR (albuminuria) was significantly higher in HIV-infected women on TDF-based ART regimen than the HIV-uninfected controls. Similarly, results from a sub-study of a randomised trial (*n* = 19) showed significant increased albuminuria in HIV-infected patients who switched to TDF-based ART regimen compared to patients who continued on a non-TDF-based ART regimen from baseline to 48 weeks follow-up.^24^ Albuminuria is an important early sign for progressive renal function loss and a significant risk factor for near-term development of overt kidney disease in HIV infection.^25^ In 85% of HIV-infected adults taking TDF-based ART, the presence of albuminuria was associated with inflammatory biomarkers.^26^ Nevertheless, the authors did not adjust for all baseline characteristics such as the use of angiotensin receptor blockers or angiotensin converting enzyme inhibitors, age and hypertension. Inflammatory biomarkers in another study^27^ were higher in untreated HIV-infected participants compared to both HIV-infected participants (TDF-based ART and virally suppressed) and uninfected controls.

Kidney tubular dysfunction (KTD) is defined when at least two of the following are present: altered resorption of phosphorus, abnormal uric acid excretion, non-diabetic glucosuria, hyperaminoaciduria or hyperphosphaturia.^12^ In a study by Rodriguez-Novoa (*n* = 92), KTD was associated with median mid-dose plasma TFV concentration of 182 ng/mL (105 ng/mL – 220 ng/mL) and normal tubular function with median mid-dose plasma TFV concentration of 106 ng/mL (75 ng/mL – 148 ng/mL).^12^ Similarly, KTD was independently associated with mid-dose TFV concentration of > 160 ng/mL in a cohort of 351 Thai participants.^14^ A study in Europe (*n* = 161) found that higher TFV plasma concentrations were associated with KTD.^15^ In this study, plasma TFV concentration was found to be a significant independent predictor of albuminuria in HIV-infected participants. The median mid-dose plasma TFV concentration associated with albuminuria was 113 ng/mL (74 ng/mL – 139.4 ng/mL). This may suggest the need for monitoring plasma TFV concentration in selected few serious cases to detect early signs of kidney tubular toxicity although it can be costly. To our knowledge, this is the first study to determine an association between plasma TFV concentration and albuminuria in HIV-infected women in South Africa. On the contrary, genetic factors in certain populations may influence renal tubular transport of TFV and contribute to the development of KTD.^28^

We did not find an association between plasma TFV concentration and eGFR or CrCl in the HIV-infected women. This is consistent with other findings^11,29^ where trough TFV concentration was used to correlate, unlike the median TFV concentration in our study, which corresponded to mid-dose. This observation is in contrast with two other studies where high plasma TFV concentrations were associated with decreased eGFR^13^ and eGFR of <90 mL/min/1.73 m^2^,^14^ respectively.

Glomerular hyperfiltration is common among HIV-infected persons. Although no common definition has been agreed upon, it varies as either abnormally high eGFR, with a threshold ranging from 125 mL/min/1.73 m^2^ to 175 mL/min/1.73 m^2^, or increased filtration fraction, or as increased filtration per nephron.^30,31^ Glomerular hyperfiltration can be an early indicator of kidney dysfunction and precedes the onset of impaired renal function and albuminuria, although the length of time it takes to progress to renal impairment is unknown.^30^ In this study, eGFR and CrCl were higher in HIV-infected women compared to the HIV-uninfected women. Furthermore, there was a higher trend in the proportion of HIV-infected participants with glomerular hyperfiltration compared to HIV-uninfected participants (33.3% vs 10%), which could be an early indicator of renal impairment. Our results are in agreement with recent findings from a prospective cohort study with a much larger sample size (*n* = 367).^30^ These authors suggested that glomerular hyperfiltration occurs due to HIV infection and ART use.

Renal function had improved in a cohort of HIV patients (*n* = 566) initiated on TDF at 6 and 12 months follow-up, with CrCl increase of +5 mL/min and +7 mL/min.^32^ In another study (*n* = 201), no significant change in eGFR from baseline value at 6, 12 and 24 months was observed in TDF exposed HIV-infected adults, mainly of African–American ethnicity.^33^ In this study, the HIV–infected participants on TDF-based ART regimen had a significant increase in CrCl and eGFR of +26.5 mL/min and +38.5 mL/min/1.73 m^2^, respectively, within a median time of 16.6 months from baseline to follow-up. A complication of HIV disease itself is reduced CrCl, and ART was found to ameliorate renal function in advanced HIV disease.^34^ ART may therefore contribute to the increase in CrCl and eGFR that we observed in HIV-infected participants, which is also in agreement with reported literature.^34^ Furthermore, a very recent retrospective study by De Waal and co-workers^35^ conducted in a very large South African cohort (*n* = 13 168 from 2010 to 2012) concluded that kidney function did recover in most patients on TDF despite having CrCl < 50 mL/min prior to TDF initiation.

Conversely, in some cohort studies, TDF therapy was associated with decreased eGFR^36,37^ that was not completely reversible.^38^ This difference could have been due to other factors such as smaller body weight and smaller BMI, which are associated with decreased eGFR in patients exposed to TDF-based ART regimen.^39^

### Limitations

This study has several limitations to consider. Firstly, the cross-sectional design only provided information at one point in time and hence could not infer causality of the relationship observed with plasma TFV and albuminuria. Secondly, the spot urine sample for the calculation of uACR was used. Thirdly, the sample size of 30 was too small to generalise the findings. Fourthly, CrCl/GFR was estimated and not measured. Despite that, the strength lays in the fact that there was the age- and BMI-matched control group (HIV-uninfected) to compare with HIV-infected participants within the same sub-study population, and various renal markers were tested at the same time in both groups for comparison. Also, there was baseline (prior to TDF treatment) information on CrCl, eGFR and SCr available to make comparisons within the HIV-infected group on TDF-based ART regimen. Our findings are nonetheless important in managing renal function monitoring in women receiving TDF-based ART regimens.

## Conclusion

In conclusion, plasma TFV concentration is independently associated with increased albuminuria in HIV-infected women within this pilot investigation. There was an increase in eGFR and CrCl in the HIV-infected women from baseline. Furthermore, longitudinal studies with larger sample sizes are needed to confirm these findings and to investigate glomerular hyperfiltration in ART-experienced patients, which may be an early sign of kidney dysfunction.
